# The Use of Dietary Supplements Among African and Caribbean Women Living in the UK: A Cross-Sectional Study

**DOI:** 10.3390/nu12030847

**Published:** 2020-03-22

**Authors:** Amanda Rodrigues Amorim Adegboye, Omorogieva Ojo, Gulshanara Begum

**Affiliations:** 1 School of Human Sciences, Faculty of Education, Health and Human Sciences, University of Greenwich, Greenwich Campus, London SE10 9LS, UK; A.Adegboye@greenwich.ac.uk; 2 School of Health Sciences, Faculty of Education, Health and Human Sciences, University of Greenwich, Avery Hill Campus, Avery Hill Road, London SE9 2UG, UK; 3 School of Life Sciences, College of Liberal Arts & Sciences, University of Westminster, Cavendish Campus, 115 New Cavendish Street, London W1W 6UW, UK; R.Begum44@westminster.ac.uk

**Keywords:** African, Caribbean, ethnic minority, dietary supplement, vitamin D, women

## Abstract

Previous studies have shown that the use of dietary supplements is associated with the prevention of birth defects, negative pregnancy outcomes and cardiovascular diseases. However, there might be some ethnic disparities in supplement usage suggesting that women who could benefit from it are not frequent users. This study aimed to characterise the use of dietary supplement among Black African and Black Caribbean women living in the United Kingdom (UK). Furthermore, it evaluated possible associations between the use of dietary supplements and health and diet awareness. A total of 262 women self-ascribed as Black African and Black Caribbean living in the UK completed a comprehensive questionnaire on socio-demographic factors, diet, use of supplements and cultural factors. The main outcome variable was the regular use of any type of dietary supplement. Use of vitamin D and/or calcium was also explored. A stepwise logistic regression analysis was applied to identify predictors of regular use of dietary supplements. A total of 33.2% of women reported regular use of any dietary supplements and 16.8% reported use of vitamin D and/or calcium. There were no significant ethnic differences in the use of dietary supplements. Reporting use of the back of food packaging label (odds ratio (OR) 2.21; 95% CI 1.07–4.55); a self-rated healthy diet (OR 2.86; 95% CI 1.19–6.91) and having cardiovascular disease (CVD), hypertension and/or high cholesterol (OR 3.81; 95% CI 1.53–9.49) increased the likelihood of using any dietary supplement. However, having poorer awareness decreased the likelihood (OR 0.94; 95% CI 0.88–0.99) of using any dietary supplement. For the use of vitamin D and/or calcium supplements, the main predictor was having CVD, hypertension and/or high cholesterol (OR 4.43; 95% CI 1.90–10.35). The prevalence of dietary supplement use was low among African and Caribbean women. Thus, awareness of potential benefits of some dietary supplements (e.g., vitamin D) among the Black population should be promoted.

## 1. Background

Ethnic minority women are at high risk of micronutrient deficiency [1]. According to Leung and Stanner [2], Black women are more likely to consume lower amounts of calcium compared to Caucasian women. In fact, 42% of Black women have been shown to have calcium intake below the Reference Nutrient Intakes (RNI) compared with 8% of Caucasians [2]. In addition, the National Institute for Health and Care Excellence (NICE) [3] has reported that people with darker skin may have a higher risk of developing vitamin D deficiency due to the fact that their skin is less efficient at synthesising vitamin D. 

In a study by Ford et al. [4], a high prevalence of vitamin D deficiency in the Black Caribbean population, especially in women, was found. The prevalence of vitamin D deficiency was 26% in Black Caribbean, 31% in Asians and 12% in Caucasians [4]. A range of factors including income and socio-economic status, food availability and access, awareness of healthy eating, religious beliefs, food beliefs and dietary acculturation may be partly responsible for food choices of Africans and Caribbean people and related vitamin D and calcium status [2]. 

The United Kingdom (UK) boasts a culturally diverse population with increasing numbers identifying as ethnic minority. In the 2011 Census, ethnic minorities accounted for almost 14% of the population in England and Wales, with African and Caribbean groups constituting 3.3% of this cohort [5]. According to Lowth [6], populations from ethnic backgrounds show differences in terms of illness behaviour, beliefs and outcomes.

The National Diet and Nutrition Survey (NDNS) found that across the UK population, average 25-hydroxyvitamin D (25-OHD) concentrations (the best indicator of vitamin D status) were lowest between January to March among all ages and genders. Approximately 19% of young children (4–10 years), 37% of older children (11–18 years) and 29% of adults were below the threshold indicating risk of deficiency (<25 nmol/L) [7]. Other work supports this by identifying at risk groups within the UK population, such as pregnant and breastfeeding women, infants, elderly and ethnic minorities [3]. Vitamin D is mainly obtained through cutaneous synthesis involving ultraviolet B sunlight irradiation and only a small contribution from the diet [8]. Clothing, use of sun lotion and increased indoor working hours may contribute to the reduced sun exposure even in tropical climates [8]. Lack of vitamin D is a major concern in the UK due to lack of sunshine and skin exposure to ultraviolet-B (UVB) light given the high level of pollution [3]. Due to its hydrophobic properties, vitamin D can be sequestered out of the blood and into adipose tissue of obese subjects and given the high prevalence of overweight and obesity among African and Caribbean women in the UK, vitamin D deficiency is a special concern [9].

In addition to the micronutrient concerns, reports have pinpointed that ethnic minority groups experience negative health outcomes disproportionately, compared to their Caucasian counterparts, an experience which is often coined as “health inequality”. The poorer health outcomes among these groups are often seen in conditions such as diabetes [9], obesity [10] and cardiovascular diseases [2]. For example, African (38%) and Caribbean (32%) women have the highest obesity prevalence among all ethnic groups in the UK [11].

Previous studies have suggested that use of certain dietary supplements may lower risk of birth defects, coronary heart disease (CHD) [12] and cardiovascular disease (CVD) [13], and can prevent negative pregnancy outcomes [14,15,16]. Research from the United States of America (USA) has suggested that there are social disparities regarding use of dietary supplements, indicating the possibility that more vulnerable populations are less likely to be aware of and take supplements when required and consequently take advantage of their potential health benefits [17].

Furthermore, there is evidence that low vitamin D status has been associated with diabetes in the general population [18], as well as gestational diabetes mellitus [19] and preeclampsia [20] among pregnant women. Vitamin D influences calcium homeostasis [14] and is useful for the growth of the skeleton and healthy bones [3]. It enhances calcium absorption in the gut, promotes normal bone mineralization and prevents hypocalcaemia [21]. Therefore, NICE [3] has recommended that Africans and Black Caribbean families (and other vulnerable groups) should increase their access to vitamin D supplements. These supplements could be in the form of vitamin D alone or as multivitamin products including calcium [3].

There is a scarcity of studies investigating factors influencing the use of dietary supplements and of those published, few have focused on ethnic minorities, their health beliefs and attitudes. The aim of this study was to characterise dietary supplement use among African and Afro-Caribbean women living in the UK. It was also to evaluate possible associations between the use of dietary supplements and health and diet awareness.

## 2. Methods

### 2.1. Study Design

This study used data from the ATTITUdinal DEterminants of diet and lifestyle among African and Caribbean women, also known as the ATTITUDE study. The ATTITUDE study applied a cross-sectional design which used a comprehensive questionnaire to capture socio-demographic data and explore general attitudes towards diet and lifestyle. The full study protocol has been described elsewhere [22]. Ethical approval was granted by the University of Westminster ethics committee under reference VRE1415-1345. All participants provided an electronic or paper-based informed consent.

### 2.2. Study Population

The study included 307 women, aged 18 years old or older, who self-identified as Black African or Black Caribbean living in London. Women reporting any severe medical condition that interfered with their dietary and physical activity behaviours were not eligible. Women who were pregnant, breastfeeding, with less than six weeks after childbirth, with a cognitive disability and/or with any disability or who were physically unable to take part in the study and who had provided informed consent were not included in the study. After exclusion of those with missing information on use of dietary supplement and main socio-demographic factors (e.g., age and education), the final study population comprised 262 women.

### 2.3. Recruitment

Although the study sample was self-selected, the researchers applied a component of purposeful sampling to ensure diversity in terms of age distribution and ethnicity. Women were recruited across London boroughs (such as Croydon, Greenwich, Enfield and Lewisham) with good representation of the Black African and Black Caribbean population. Women were invited to take part in the study via emails, social media, advertisements, flyers and posters distributed in community hubs, places of worship, university campuses and via liaison with church leaders. Women were also opportunistically recruited (face-to-face) from community settings (leisure centres and libraries) and via snowballsampling. Recruitment of participants was completed in October 2017.

### 2.4. Data Collection

The data collection process included three stages. The first stage (stage 1) included a self-administered questionnaire. Those who completed the questionnaire were invited to book a face-to-face visit and take part in stage 2. Stage 2 involved objective measures of biochemical and anthropometric indices, blood pressure, pulse and heart rate in a subsample of the participants who completed the questionnaire. The final stage (stage 3) included a dietary assessment via repeated 24-h dietary recall for the same participants in stage 2. In the current study, only data from stage 1 were presented and analysed.

Participants filled in an online questionnaire, designed in Qualtrics software (Qualtrics, Provo, Utah, USA). The online questionnaire was piloted prior to the data collection and tested on iPhones, androids, desktops using different software and hardware configurations. The quick response (QR) code and a uniform resource locator (URL) link to the questionnaire were distributed via social media, study website, email and on flyers and posters. However, it was not possible to identify if the questionnaire link was accessed via social media, email, flyers or posters. A paper-based questionnaire was also available upon request. Data obtained from manual completion of the questionnaire were entered into the survey software tool to generate a joint database for data analysis. Less than 10% of the participants used the paper-based questionnaire.

The online questionnaire included a brief study description and three eligibility questions: (1) “Are you female?”, (2) “Are you aged 18 or older?” and (3) “Are you of Black British, Black African or Black Caribbean ethnicity?” Only those who answered yes to all three questions were deemed eligible to complete the questionnaire. Thereafter, participants were asked to indicate (yes/no) if they consented to take part in the study and share their nonidentifiable data for research purposes.

The questionnaire was composed of 83 questions, which were subdivided into six sections: (1) demographic data (including migrant history and country of birth), self-rated health, education and socio-economic status; (2) diet-related knowledge and attitudes; (3) shopping and understanding and use of food labels; (4) self-reported body weight and height, body image and shape; (5) physical activity and (6) ethnicity, cultural affiliation, language spoken at home and religion. Participants were asked to state their country of birth and self-identify their ethnicity as Black African, Black Caribbean and mixed Black. The mixed Black group included white and black or African and Caribbean.

Self-rated health status in the last 3 months is considered a reliable measure of health and an important predictor of morbidity and mortality and valid for different ethnic groups [23,24]. Self-rated health was measured on a four-point scale (excellent, good, fair and poor) as reported by Baruth et al. [25]. For the purpose of analyses, the categories were merged to create a binary variable (excellent/good vs. fair/poor). The survey also included a question whether participants were diagnosed with a list of seven different conditions (e.g., CVD, diabetes, hypertension, high cholesterol, asthma, arthritis and cancer). Participants could select all conditions applicable and they also had an option to add any additional condition not listed. Self-rated dietary quality question, classified as “very healthy”, “quite healthy”, “not very healthy” and “very unhealthy”, was based on the Health Survey for England (HSE) [26].

Awareness and valuation of healthy eating was assessed via a range of questions from the 2007 HSE [26]. Participants were asked to rate how important 13 different statements were as part of healthy eating habits (such as limiting salt, eating lots of fruits and vegetables and taking vitamin supplements) in a five-point Likert scale ranging from very important to not at all important and cannot choose. The Likert scale variable was converted into a numeric variable by assigning a score for each category. The scale including all 13 statements ranged from 13 (high awareness) to 65 (low awareness) of factors influencing a healthy dietary habit. Participants were also asked how often they usually use labels on the back of food packaging to find out how healthy a food product is. The response options were always, often, sometimes, rarely and never.

### 2.5. Data Analysis

The main dependent variable was the use of any dietary supplements (yes/no). The use of dietary supplements was considered if participants reported intake of any type of supplement regardless of dose, brand, frequency and duration. Additionally, the use of vitamin D and/or calcium supplement was investigated. The inclusion of calcium supplement users together with vitamin D users was due to the fact that most calcium supplements also include a modest amount of vitamin D as it is essential for calcium absorption. When it came to the different health conditions, hypertension, cardiovascular disease and high cholesterol were grouped into one single category (yes/no). For the purpose of analyses, the self-rated diet quality categories were merged to create a binary variable (very healthy/quite healthy vs. not very healthy/very unhealthy). Similarly, for analysis purposes, the categories for frequency of use of food labels were merged into two categories (always and often vs. sometimes, rarely and never).

The chi-squared test and independent *t*-test were used for comparison between dietary supplement use and categorical (%) and continuous (means) variables, respectively. Multiple logistic regressions were used to explore factors associated with the use of any type of dietary supplement and use of vitamin D and/or calcium supplement. A backward stepwise approach was applied to identify the best fit model. All predictors which showed a *p*-value below or equal to 0.1 in the bivariate analysis was included in the stepwise regression. The probability of removing the option from the full model was set to 0.1. Statistical analyses were performed using STATA (Stata TX, version 15, Texas, USA).

## 3. Results

The study population of 262 women consisted of 166 (63.4%) Black African, 71 (27.1%) Black Caribbean and 25 (9.5%) mixed Black. Around 54.4% of the study population were born outside of the UK. Among those not born in the UK, the majority were born in West Africa (44.2%), followed by the Caribbean (19.4%), South Africa (14%) and East Africa (13.2%), and only a minority were born in Europe, South America or North America (9.2%). The most frequent countries of birth were Nigeria, Ghana and Jamaica.

The mean age was 34 years (SD 10.8). The youngest participant was 18 years old and the oldest was 65 years old; 88.5% were considered of childbearing age (up to 49 years-old) and 46.2% had children. In terms of education and employment, 75.6% and 71% of the population reported having post-secondary education and being employed, respectively. The mean body mass index (BMI) based on self-reported weight and height was 29.6 kg/m^2^ (SD 7.5).

In total, 87 out of 262 women (33.2%) reported regular use of dietary supplements and 44 (16.8%) reported use of vitamin D and/or calcium supplements. The majority of women who reported use of calcium supplement (12 out of 13) also reported the use of vitamin D. Only one participant reported use of calcium supplement exclusively. However, most of the calcium supplement contains a small to moderate amount of vitamin D and therefore these two groups were combined. Among those who reported supplement use (*n* = 87), the most frequently used supplements were multivitamins (23.9%) followed by vitamin D (19.7%) (see Table 1 for more details). Calcium and other types of supplements were the least frequent categories. The “other” category included turmeric, zinc, magnesium, selenium and biotin. Only two women reported use of folic acid. Some of the participants also included more than one choice of dietary supplements. There were no significant differences in the frequency of most commonly used supplements across ethnic groups (full data presented in Table 1).

Table 2 shows the general characteristics of the study population according to use of any dietary supplements. Women who reported use of dietary supplements were significantly older (*p* = 0.033) and more likely to be aware of factors influencing a healthy diet pattern (measured by the awareness score); have post-secondary education; report a healthy diet; use the back of food packaging label and have CVD, hypertension and/or high cholesterol compared to those who did not report the use of dietary supplements. When investigating the differences between users and non-users of vitamin D and/or calcium specifically, it was observed that users also tended to be older and were more likely to report a healthy diet; use the back of food packaging label and have CVD, hypertension and/or high cholesterol compared to non-users of vitamin D and/or calcium supplements (Table 3).

In the multivariate logistic regression models, a few variables were considered significant predictors of use of any dietary supplements or use of vitamin D and/or calcium supplements (Table 4). Reporting use of the back of food packaging label, a self-rated healthy diet and having CVD, hypertension and/or high cholesterol significantly increased the odds of using any dietary supplement. However, having poor dietary awareness on the basis of the healthy diet awareness score decreased the odds of using any dietary supplement. Although only few variables were retained in the final model and pseudo *R^2^* was low (9.4%), the model fit was statistically significant (*p*-value < 0.0001), which tells that the model as a whole fits significantly better than an empty model.

For the use of vitamin D and/or calcium supplement model, the main predictor was having CVD, hypertension and/or high cholesterol. Report of having a healthy diet increased the odds of use of vitamin D and/or calcium supplement; however, the *p*-value was borderline (*p* = 0.053) (Table 4). Although pseudo *R^2^* for this model was low (7%) the final model was statistically significant (*p*-value = 0.0003).

## 4. Discussion

The current study showed that the use of dietary supplement (33.2%) was low among African and Caribbean women living in the UK compared to studies using similar and other communities. In the multivariate model, diagnosis of CVD, hypertension and/or high blood pressure; self-rated diet quality and use of food label significantly predicted the likelihood of dietary supplement usage. Previous studies in the USA [17,27,28] showed a higher prevalence of supplement use and that prevalence varied markedly according to socio-demographic factors, which is consistent with the hypothesis that the most vulnerable groups that might most benefit from the use of dietary supplements are those that consume or access them the least. In 2003, a study (which was based in the USA) by Jasti et al. [17] recruited a total of 2868 women aged 18 years or older who completed the Diet and Health Knowledge (DHK) Survey questionnaire [17]. These authors reported that Caucasian women (57%) had the highest prevalence of use of supplements, followed by Hispanic women (45%), women from other ethnicities (45%) and Black women (40%) [17]. Although Black women had the lowest prevalence of supplement use, the proportion of Black women was much higher than the prevalence observed in the current study. In a study conducted in the UK, it was found that 48% of the study population reported current use of dietary supplements. However, information on use among minority groups was not provided [29]. A Canadian Community Health Survey (CCHS) also reported a higher prevalence of supplement use (40.1%) in the general population of Canadians [30] compared to the findings in this study.

In the current study, the majority of the population were within childbearing age (up to 49 years old). Some dietary supplements are of particular relevance during pregnancy and lactation, such as vitamin D and calcium, iron and folic acid. For example, pregnancy and lactation are periods of additional calcium demand as maternal calcium is transferred to the growing foetal skeleton and for breast-milk production [14]. Consumption of calcium and vitamin D is often encouraged, particularly during pregnancy and lactation, in order to preserve maternal skeletal calcium stores, which are depleted during these two critical periods [31]. Preconception use of folic acid supplements is recommended to prevent neural tube defects [32]. Furthermore, during pregnancy, there is an increase in iron requirements due to a rise in the red blood cell mass and foetal and placental growth [33]. Consumption of these micronutrients, as supplements, were low in the current study. However, pregnant and lactating women were not eligible for this study and this may explain the low intake of such supplements in the current population.

Consistent with previous studies [17,34] supplement users were more likely to have been diagnosed with a disease and read food labels more often than non-users. Additionally, the findings from the current study show that supplement users were more likely to perceive their diet as being very or quite healthy compared to non-users. This is similar to the findings of a study carried out by van der Horst and Siegrist [35]. Using a sample of 6189 respondents who completed the Swiss Food Panel (SFP) questionnaire, van der Horst and Siegrist [35] investigated whether people use vitamin and mineral supplements to compensate for unhealthy diets, or people who already have a healthy diet use more supplements. The authors performed a cluster analysis that revealed three clusters: healthy diet, unhealthy diet and modest diet. Compared to non-users, a higher percentage of supplement users were categorised in the healthy cluster [35].

It is important to highlight that African and Caribbean are two distinct groups and they do not necessarily share the same health knowledge and attitudes towards diet and use of supplements. Interestingly, the prevalence of supplement use and the list of most commonly used dietary supplements did not differ among Black African, Black Caribbean and Mixed Blacks. However, it is essential to note that the use of ethnicity variable in the model does not explain the relevance of cultural factors regarding health beliefs and use of supplements. Therefore, other factors such as language spoken at home, religion, and country of birth were also explored, but none of these variables were associated with the use of dietary supplements in this study population.

The interpretation of these findings should take into consideration some study limitations. Detailed information of dose and frequency of supplement use were not available and study sample size was modest. Women of African and Caribbean origin should not be categorised as one single group as both Caribbean and Africans are made up of various territories with diverse cultures, languages and histories. Hence, it is not possible to assume that a small sample of women from few Caribbean and African communities may represent the perspectives of these groups. However, this study applies a more meaningful classification of ethnicity compared to the previous studies [36] in which no distinction was made between Black Africans, Black Caribbeans and other Black groups. Additionally, the population in the current study was highly educated and more than 70% reported being employed. Therefore, the prevalence of supplement use might be lower among less educated and less affluent Black women.

Over the years, the cultural diversity of the UK’s population has been increasing. Across England and Wales, the most ethnically diverse area, at present, is London [6]. Much work has identified that Black and minority ethnic (BME) groups within UK communities experience health inequalities [2,11]. A better understanding of the UK’s ethnic mix can help to improve healthcare delivery using targeted and connected programmes [6]. The ATTITUDE study is a cross-sectional quantitative study that included a comprehensive questionnaire among an understudied population of African and Caribbean women in the UK. A quantitative study can provide relevant information on contextual influences and determinants of health-related behaviour including the use of dietary supplements. Understanding these influences will inform the theory underpinning the design of culturally tailored interventions and consequently supporting these women.

## 5. Conclusions

Use of dietary supplements, during certain times in life, offers individuals the ability to play an active role in their own health and well-being. Although previous research has shown that around half of UK adults currently take supplements on a regular basis, little is mentioned about whether any variations of supplement use are present between ethnicities. Much work on supplement use in multi-ethnic cohorts has been carried out but few studies within UK populations. To the best knowledge of the authors, the current study, appears to be one of the few exploring overall supplement use within a cohort of Black African/Caribbean women in the UK. Within this group, poor dietary awareness decreased the odds of using of dietary supplements. Furthermore, few women reported consumption of vitamin D, calcium, iron and folic acid—vital supplements at particular time points in life. Steps need to be taken to better understand the intricacies with respect to supplement use within multi-ethnic communities in order to have conversations and incorporate learning opportunities to prevent misuse and encourage proper use.

## Figures and Tables

**Table 1:** Frequently used supplements among those who reported any use of dietary supplements by ethnic group (*n* = 87).

Supplements *	Black African *n* (%)	Black Caribbean *n* (%)	Mixed- Black *n* (%)	Total *n* (%)
Multivitamins	32 (61.5)	11 (21.2)	9 (17.3)	52 (23.9)
Vitamin D	30 (69.8)	9 (20.9)	4 (9.3)	43 (19.7)
Vitamin C	22 (61.1)	10 (27.8)	4 (11.1)	36 (16.5)
Omega 3	19 (57.6)	8 (24.2)	6 (18.2)	33 (15.1)
Iron	17 (68)	7 (28)	1 (4)	25 (11.5)
Other	7 (43.8)	7 (43.8)	2 (12.4)	16 (7.3)
Calcium	8 (61.5)	4 (30.8)	1 (7.7)	13 (6)

* *p*-value for Pearson exact chi-squared test = 0.939 (non-significant difference across groups). Note: multiple responses were allowed. This constitutes 218 answers in 87 cases.

**Table 2:** Use of any dietary supplements according to general characteristics.

	Dietary Supplement Use			*p*-Value
	Yes	No	Total	
	Mean (SD)	Mean SD)	Mean (SD)	
Age (years) *	36 (10.5)	33 (10.9)	34 (10.8)	0.033
BMI (kg/m^2^) *	26.7 (6.1)	30 (8.1)	29.6 (7.5)	0.191
Healthy diet awareness (score) *	23.2 (4.7)	25.2 (5.4)	24.6 (5.3)	0.005
	***n* (%)**	***n* (%)**	***n* (%)**	
**Ethnicity**				
Black African	52 (59.8)	114 (65.1)	166 (63.4)	0.452
Black Caribbean	24 (27.6)	47 (26.9)	71 (27.1)	
Mixed Black	11 (12.6)	14 (8)	25 (9.5)	
**Post-secondary education**				
Yes	73 (83.9)	125 (71.4)	198 (75.6)	0.027
No	14 (16.1)	50 (28.6)	64 (24.4)	
**Employment status**				
Employed	63 (72.4)	123 (70.3)	186 (71.0)	0.721
Unemployed or retired	24 (27.6)	52 (29.7)	76 (29.0)	
**Civil status**				
Married or living with a partner	30 (34.5)	58 (33.1)	88 (33.6)	0.829
Single, divorced or widow	57 (65.5)	117 (66.9)	174 (66.4)	
**English the only language spoken at home**				
Yes	40 (46.0)	68 (38.9)	108 (41.2)	0.270
No	47 (54.0)	107 (61.1)	154 (58.8)	
**Self-rated health status**				
Excellent or good	18 (20.7)	44 (25.1)	62 (23.7)	0.424
Fair or poor	69 (79.3)	131 (74.9)	200 (76.3)	
**Diagnosis of CVD, hypertension and/or high cholesterol**				
Yes	158 (92.9)	70 (81.4)	228 (89.1)	0.005
No	12 (7.1)	16 (18.6)	28 (10.9)	
**Self-rated diet quality**				
Very or quite healthy	77 (88.5)	128 (73.1)	205 (78.2)	0.005
Not very healthy or unhealthy	10 (11.5)	47 (26.9)	57 (21.8)	
**Use of label on the back of the food packaging**				
Yes	70 (81.4)	121 (69.1)	191 (73.2)	0.036
No	16 (18.6)	54 (30.9)	70 (26.8)	
**Having a child**				
Yes	42 (48.3)	79 (45.1)	121 (46.2)	0.632
No	45 (51.7)	96 (54.9)	141 (53.8)	

Body Mass Index (BMI); Cardiovascular disease (CVD); *continuous variables presented as mean and standard deviation (SD).

**Table 3:** Use of vitamin D and/or calcium supplements according to general characteristics.

	Dietary Supplement Use			*p*-Value
	Yes	No	Total	
	Mean (SD)	Mean (SD)	Mean (SD)	
Age (years) *	37.4 (11.7)	33.3 (10.5)	34 (10.8)	0.022
BMI (kg/m^2^) *	29.6 (6.6)	29.6 (7.6)	29.6 (7.5)	0.100
Healthy diet awareness (score) *	23.5 (4.8)	24.8 (5.3)	24.6 (5.3)	0.153
	***n* (%)**	***n* (%)**	***n* (%)**	
**Ethnicity**				
African	30 (68.2)	136 (62.4)	166 (63.4)	0.766 **
Caribbean	10 (22.7)	61 (28)	71 (27.1)	
Mixed Black	4 (9.1)	21 (9.6)	25 (9.6)	
**Post-secondary education**				
Yes	37 (84.1)	161 (75.8)	198 (75.6)	0.180 **
No	7 (15.9)	57 (26.1)	64 (24.4)	
**Employment status**				
Employed	36 (81.8)	150 (68.8)	186 (71)	0.08
Unemployed or retired	8 (18.2)	68 (31.2)	76 (29)	
**Civil status**				
Married or living with a partner	15 (35.1)	73 (33.5)	88 (33.6)	0.938
Single, divorced or widow	29 (65.9)	145 (66.5)	174 (66.4)	
**English the only language spoken at home**				
Yes	18 (40.9)	90 (41.3)	108 (41.2)	0.963
No	26 (59.1)	128 (58.7)	154 (58.8)	
**Self-rated health status**				
Excellent or good	34 (77.3)	166 (76.2)	200 (76.3)	0.873
Fair or poor	10 (22.7)	52 (23.8)	62 (23.7)	
**Diagnosis of CVD, hypertension and/or high cholesterol**				
Yes	12 (27.3)	16 (7.6)	28 (10.9)	<0.0001
No	32 (72.7)	196 (92.4)	228 (89.1)	
**Self-rated diet quality**				
Very or quite healthy	40 (90.9)	165 (75.7)	205 (78.2)	0.027 **
Not very healthy or unhealthy	4 (9.1)	53 (24.3)	57 (21.8)	
**Use of label on the back of the food packaging**				
Yes	36 (81.8)	155 (71.4)	191 (73.2)	0.160
No	8 (18.2)	62 (28.6)	70 (26.8)	
**Having a child**				
Yes	23 (52.3)	98 (44.9)	121 (46.2)	0.374
No	21 (47.7)	120 (55.1)	141 (53.8)	

Body Mass Index (BMI); Cardiovascular disease (CVD); * continuous variables presented as mean and standard deviation (SD); ** Fisher exact test.

**Table 4:** Multiple logistics model for predictors of use of any dietary supplements and use of vitamin D and/or calcium supplement.

**Use of Any Dietary Supplements**			
**Predictors**	OR *	**95% CI**	***p*-Value**
Diagnosis of CVD, hypertension and/or high cholesterol	3.81	1.53–9.49	0.004
No diagnosis of CVD, hypertension and/or high cholesterol	1 (reference)		
Very or quite healthy self-rated diet quality	2.86	1.19–6.91	0.019
Not very healthy or unhealthy self-rated diet quality	1 (reference)		
Healthy diet awareness (score) **	0.94	0.88–0.99	0.029
Use of label on the back of the food packaging	2.21	1.07–4.55	0.031
No use of label on the back of the food packaging	1 (reference)		
**Use of Vitamin D and/or Calcium Supplement**			
**Predictors**	**OR**	**95% CI**	***p*-Value**
Diagnosis of CVD, hypertension and/or high cholesterol	4.43	1.90–0.3	0.001
No diagnosis of CVD, hypertension and/or high cholesterol	1 (reference)		
Very or quite healthy self-rated diet quality	2.94	0.98–8.76	0.053
Not very healthy or unhealthy self-rated diet quality	1 (reference)		

Cardiovascular disease (CVD); * Adjusted odds ratio (OR). The multivariate analysis included all predictors presented in the table; ** continous variable, therefore the adjusted OR represent the odd of the outcome of each unit increase on the scale.
